# An Energy Harvester with Temperature Threshold Triggered Cycling Generation for Thermal Event Autonomous Monitoring

**DOI:** 10.3390/mi12040425

**Published:** 2021-04-13

**Authors:** Ruofeng Han, Nianying Wang, Qisheng He, Jiachou Wang, Xinxin Li

**Affiliations:** 1 State Key Laboratory of Transducer Technology, Shanghai Institute of Microsystem and Information Technology, Chinese Academy of Sciences, Shanghai 200050, China; hanruofeng@mail.sim.ac.cn (R.H.); wangny@mail.sim.ac.cn (N.W.); heqisheng2018@168.com (Q.H.); jiatao-wang@mail.sim.ac.cn (J.W.); 2 School of Microelectronics, University of Chinese Academy of Sciences, Beijing 100049, China; 3 School of Information Science and Technology, ShanghaiTech University, Shanghai 201210, China

**Keywords:** energy harvester, temperature threshold, piezoelectricity, vibrational cantilever, bimetallic effect

## Abstract

This paper proposes a temperature threshold triggered energy harvester for potential application of heat-event monitoring. The proposed structure comprises an electricity generation cantilever and a bimetallic cantilever that magnetically attract together. When the structure is heated to a pre-set temperature threshold, the heat absorption induced bimetallic effect of the bimetallic cantilever will cause sufficient bending of the generation cantilever to get rid of the magnetic attraction. The action triggers the freed generation cantilever into resonance to piezoelectrically generate electricity, and the heated bimetallic cantilever dissipates heat to the environment. With the heat dissipated, the bimetallic cantilever will be restored to attract with the generation cantilever again and the structure returns to the original state. Under continual heating, the temperature threshold triggered cycle is repeated to intermittently generate electric power. In this paper, the temperature threshold of the harvester is modeled, and the harvester prototype is fabricated and tested. The test results indicate that, with the temperature threshold of 71 °C, the harvesting prototype is tested to generate 1.14 V peak-to-peak voltage and 1.077 μW instantaneous power within one cycle. The thermal harvesting scheme shows application potential in heat event-driven autonomous monitoring.

## 1. Introduction

Wireless sensor networks (WSN) need a huge number of sensing nodes for unattended monitoring to undesired events like industrial equipment failure, human health problem, and environmental disaster, etc. [1]. Many sensor nodes working in the field suffer the common problem of insufficient on-site power supply. For example, in unattended application of forest fire monitoring, field power supply including battery replacement is really difficult. By using on-site energy harvesters, the generated electric power from environment is still often too weak to sustain the whole sensor-node microsystem which includes the sensor, the circuits for signal processing and event identification and the alarming device, et al. Inspired by the event driven analogue-to-information (A-to-I) concept [2], an energy-to-information (E-to-I) idea has arisen in our minds, where the event information of forest firing is obtained simultaneously with the electric-energy converted from the fire induced heat. Among the environmental energies such as solar energy [3], vibration energy [4] and thermal energy [5], thermal energy is one of the richest energy resources that can be converted into electricity.

The common converting mechanism from thermal energy includes thermoelectricity, pyroelectricity, and thermomagneticity [6]. Thermoelectricity is a conversion mechanism based on the Seebeck effect; that is, when two dissimilar electrical conductors or semiconductors are joined together, they make a thermocouple and if the temperature difference is maintained between the two joining junctions, an electromotive force is developed [7,8]. Hao et al. proposed a high efficiency thermoelectric energy harvester with the working temperature between 100 °C and 300 °C by suppressing intrinsic excitation in p-type Bi_2_Te_3_-based materials [9]. Pyroelectric conversion originates from interaction between polarization and temperature change in some dielectric materials [10]. Leng et al. designed a pyroelectric generator based on a polyvinylidene fluoride film to harvest the heat energy from hot/cold water [11]. The device achieves practical application by simply alternating contact with hot flow and cold flow. Thermomagnetic conversion mechanism relies on the effect of heat on the magnetic properties [12]. Chun et al. reported a thermo-magneto-electric generator array that is composed of flexible polyvinylidene difluoride bimorph cantilevers [13]. Under thermal gradient, the ferromagnetic phase transition of soft magnet generates vibration and piezoelectric power. Deepak et al. developed a novel hybrid thermomagnetic oscillator by using (MnNiSi)_0.7_(Fe2Ge)_0.3_ as thermomagnetic material for cooling of the heat load as well as electricity harvesting [14]. The proposed thermomagnetic oscillator can cool the heat load by mechanical oscillation between the load and thermal sink by up to 70 °C. Song et al. developed a magneto-thermal generator by using La_0.85_Sr_0.15_MnO_3_ and (Ni_0.6_Cu_0.2_Zn_0.2_)Fe_2_O_4_ composite material. The fabricated magneto-thermal generator shows 0.2 Hz operation frequency and generated maximum outputs of 17 μW under the thermal gradient of 80 °C [15]. Waske et al. developed a thermomagnetic generating concept to convert low-temperature waste heat into electricity. Through a combination of experiment and simulation, it is shown that the pretzel-like topology results in a sign reversal of the magnetic flux, which makes the output voltage and power of the proposed generator two orders of magnitude larger than those in conventional set-ups [16].

In contrast to the above-mentioned methods, we herein propose a thermal energy harvester with a thermo-mechanical-electrical conversion mechanism. The proposed energy harvester is composed of a generation cantilever and a bimetallic cantilever and uses the bimetallic effect of the bimetallic cantilever to achieve heat event-driven cycling energy harvesting. The temperature threshold of the energy harvester can be preset by adjusting the distance between the generation cantilever and the bimetallic cantilever. In the following sections, a technical description of the working principal, threshold design, and experimental results of the proposed energy harvester is given exhaustively.

## 2. Design and Modeling

### 2.1. Working Principle

**Figure 1:**
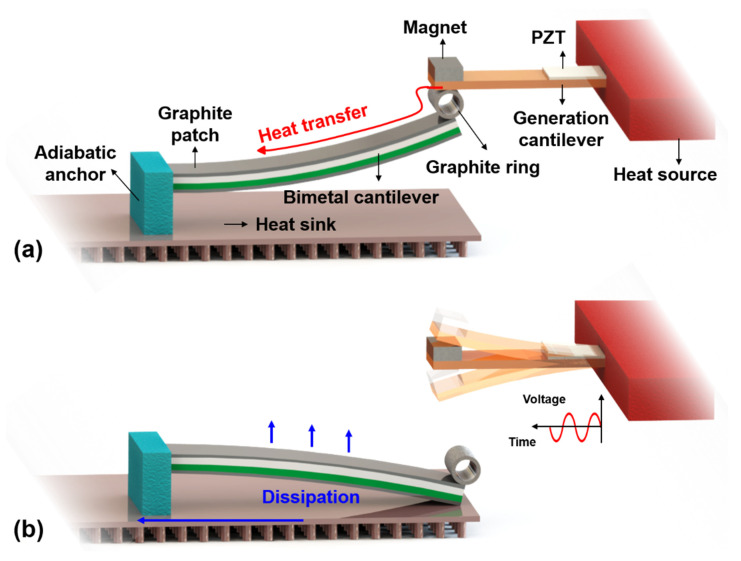
Schematic of the energy harvester for autonomous heat monitoring. (**a**) Endothermic state where the generation cantilever and the bimetallic cantilever are attracted; (**b**) exothermic state where the bimetallic cantilever and the generation cantilever are separated, and the freed generation cantilever is vibrating and generating electricity.

The energy harvester consists of two miniature cantilevers that are electricity generation cantilever (GC) and bimetallic cantilever (BC). With lead zirconium titanate (PZT) piezoelectric film coated for generation, the highly thermo-conductive generation cantilever is magnetically coupled with the temperature sensitive bimetallic cantilever to realize a temperature threshold triggering effect, where the magnetized bimetallic cantilever is attracted to the end of the generation cantilever. The metal generation cantilever can be used to absorb or conduct heat from a heat source like fire or high temperature to cause spontaneous combustion. As shown in Figure 1a, the bimetallic cantilever is originally attracted into contact with the generation cantilever via an anchored graphite ring for flexible contact and good heat conductivity. The ferromagnetic bimetal structure of the bimetallic cantilever consists of two alloy layers with different thermal expansion coefficients. Graphite sheets are attached to the double surfaces of the bimetallic cantilever to enhance thermal transfer in endothermic process and heat radiation in exothermic process.

After the generation cantilever absorbs sufficient heat from the monitored object, the temperature of the bimetallic cantilever will rise quickly due to the high thermal conductivity of graphite. Then the bimetallic cantilever will bend downwards due to bimetallic effect, thereby causing deflection of the generation cantilever. The more the absorbed heat, the larger the deflection of the generation cantilever. When the deflection induced elastic force is greater than the magnetic force, the generation cantilever will get rid of the bimetallic cantilever and freely resonates to generate electricity with the piezoelectric film, as shown in Figure 1b. At this stage, the generation cantilever is in the state of end-free beam for free resonating power-generation. The critical temperature point of the heating induced separation between the generation cantilever and the bimetallic cantilever is defined as a temperature threshold. At the temperature threshold, the bimetallic cantilever is separated from the heat source and its bending causes contact with the heat sink metal plate placed below the bimetallic cantilever, and the heat in the bimetallic cantilever will be dissipated not only by thermal convection and radiation with environment but also through the contact with the heat sink. With the loss of heat, the bimetallic cantilever will gradually recover to its original position and be attracted to the generation cantilever. Then the heat dissipation induced bimetallic restoration of the bimetallic cantilever will cause the generation cantilever to vibrate and generate electricity again.

During the endothermic process induced electric harvesting, the generation cantilever is separated from the bimetallic cantilever, and the end-free cantilever vibrates to generate electricity. In contrast, during the exothermic process induced harvesting, the generation cantilever and the bimetallic cantilever are attracted together to form a double-clamped beam like vibrating structure. Therefore, the end-free cantilever features larger resonating amplitude and higher electricity-power generation compared to the double-clamped like structure, and the resonant frequency of the end-free cantilever is lower than that of the double-clamped like one. Thus, the power generation process caused by separation of the two cantilevers is called "strong harvest", and the generation caused by attraction between the two cantilevers is called "weak harvest". The generation cycle can be repeated to generate electric power and the electric generation itself signifies that the monitored heat event occurs, i.e., the heat induced temperature on the generation cantilever exceeds the temperature threshold that can be preset by specifically designing the structure.

### 2.2. Thermal Simulation of the Bimetallic Cantilever

According to the working principle of the proposed energy harvester, the response time of the energy harvester to temperature is quite dependent on the time required for the bimetallic cantilever to separate from the generation cantilever when heated, that is, the rate of heat conduction on the bimetallic cantilever. The response cycle of the system also depends heavily on the time required for the bimetallic cantilever to return to the original position after being separated from the heat source, that is, the heat dissipation rate of the bimetallic cantilever. Therefore, in order to improve the response rate of the entire system, this research mainly focuses on the effect of the temperature response rate of some attached materials that can be used for heat conduction/heat dissipation on the bimetallic cantilever.

There are three ways of heat transfer [1]: heat conduction, heat convection, and heat radiation. For this device, heat conduction plays a significant role in the process of the bimetallic cantilever absorbing heat to separate from the generation cantilever, because the better the heat conduction performance, the faster the bimetallic cantilever responds to the heat event. According to Fourier’s Law expressed as Equation (1), it can be known that the performance of heat conduction is related to the thermal conductivity κ of the thermally conductive material, the surface area *A* crossed by the heat flux *Q*, the thickness *e* of the heat transfer direction, and the temperature difference ΔT. Thermal conductivity is a coefficient related to the properties of the material itself which means under the same operating conditions, the thermal conductivity mainly depends on the properties of the material itself.

(1) $$Q=\kappa \times A\times \frac{\Delta T}{e}\;$$

According to the Newton’s law of cooling expressed as Equation (2), the heat convection mainly depends on the temperature difference Δ*T* and the convective heat transfer coefficient *h_c_*. The convective heat transfer coefficient mainly depends on the surface condition of the material and the physical properties of fluid. When the environmental conditions remain unchanged and the surface conditions of different thermal conductive materials are relatively consistent, *h_c_* can be regarded as a constant.

(2) $$Q=h_{c}\times A\times \Delta T\;$$

Regarding the heat of thermal radiation, it can be analyzed by the Stefan–Boltzmann law shown in Equation (3):

(3) $$Q=\varepsilon \times \sigma \times A\times \left(T^{4}-T_{a}{}^{4}\right)\;$$

where *ε* is emissivity of the material, σ is blackbody radiation constant which equals to 5.67 × 10^−8^ W/(m^2^·K^4^), and *T_a_* is ambient temperature. From Equation (3) we can see that thermal radiation capacity not only depends on the emissivity but also heavily on the temperature of the object, since the exponent of temperature is the fourth power. The emissivity, similar to the convective heat transfer coefficient, is also a physical quantity related to the surface condition of the object. Therefore, it is necessary to find a kind of material with good thermal conductivity and excellent performance in the heat dissipation.

Due to the poor thermal conductivity of the bimetallic beam itself, as shown in Table 1, this study aimed at several common heat conductive materials on the market, such as graphite sheets, copper foils, and liquid alloy gallium-based alloys for simulation by using the Multiphysics software COMSOL. Table 1 shows the thermal conductivity and the emissivity of the aforementioned materials compared with the bimetal we used in the experiment.

**Figure 2:**
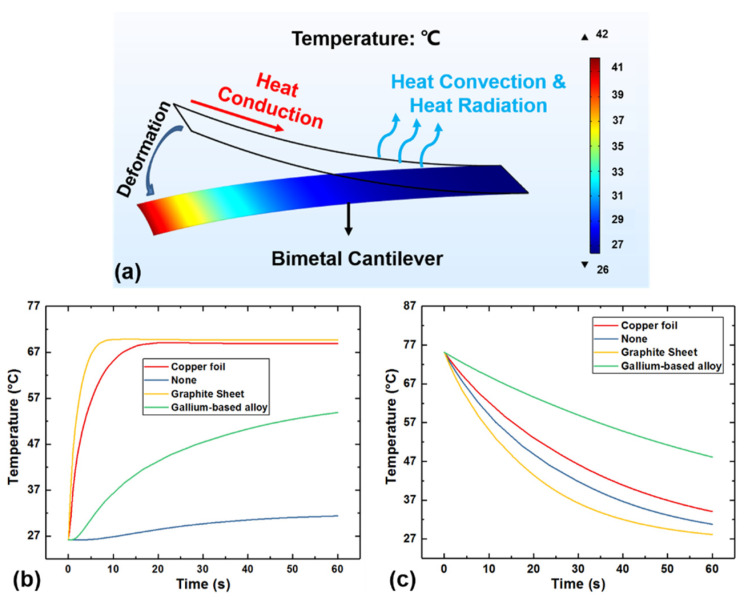
Heat conduction/dissipation simulation of the bimetallic cantilever. (**a**) Schematic diagram of the thermal deformation of the bimetallic cantilever; (**b**) the temperature curves when attaching different thermal conductive materials; and (**c**) the heat dissipation curves when attaching different thermal conductive materials.

The result of the simulation is shown in Figure 2. Figure 2a is a schematic diagram of the bimetallic cantilever after one end is fixed and the other end is deformed due to heat. The bimetallic cantilever has an initial curvature (8.33/m) at room temperature, so when one end is fixed, the other end will have an initial deflection. Figure 2b shows the transient heat conduction simulation when different thermal conductive materials are attached to the surface of the bimetallic cantilever. It can be seen from Figure 2b that the bimetallic cantilever with graphite sheets attached takes the shortest time to reach the highest steady-state temperature while the bimetallic cantilever without any thermal conductive materials attached has the longest heating time for the temperature to rise to its steady-state temperature, which indicates that attaching heat conductive materials can increase the thermal conductivity and steady-state temperature of the bimetallic cantilever. Figure 2c shows the simulation of heat dissipation of the bimetallic cantilever after it is separated from the heat source. It can be seen from Figure 2c that the bimetallic cantilever with graphite sheets still has the fastest heat dissipation rate, and the bimetallic cantilever without attaching any thermal conductive material has the second highest heat dissipation rate. The main reason for this phenomenon is that the attachment of the thermal conductive material increases the thermal resistance of the bimetallic cantilever when dissipating heat, which makes the heat dissipation capacity weaker than that of the no material attached situation. At the same time, the emissivity of the graphite sheet is much higher than the other thermal conductive materials, so the final heat dissipation capacity of the bimetallic cantilever with graphite sheet attached is the best.

### 2.3. Temperature Threshold Simulation

Since the low thermal expansion layer of the bimetallic cantilever is Invar alloy with nickel content of 36%, the bimetallic cantilever can be magnetized by the permanent magnet at the end of the generation cantilever. Therefore, the temperature threshold of the entire device can be set by the magnetic coupling between the two cantilevers, that is, the stronger the magnetic force, the higher the temperature threshold. The magnetic force between the generation cantilever and the bimetallic cantilever can be adjusted by the distance between the two stages. Table 2 shows the material properties and geometric parameters of the designed energy harvester.

**Figure 3:**
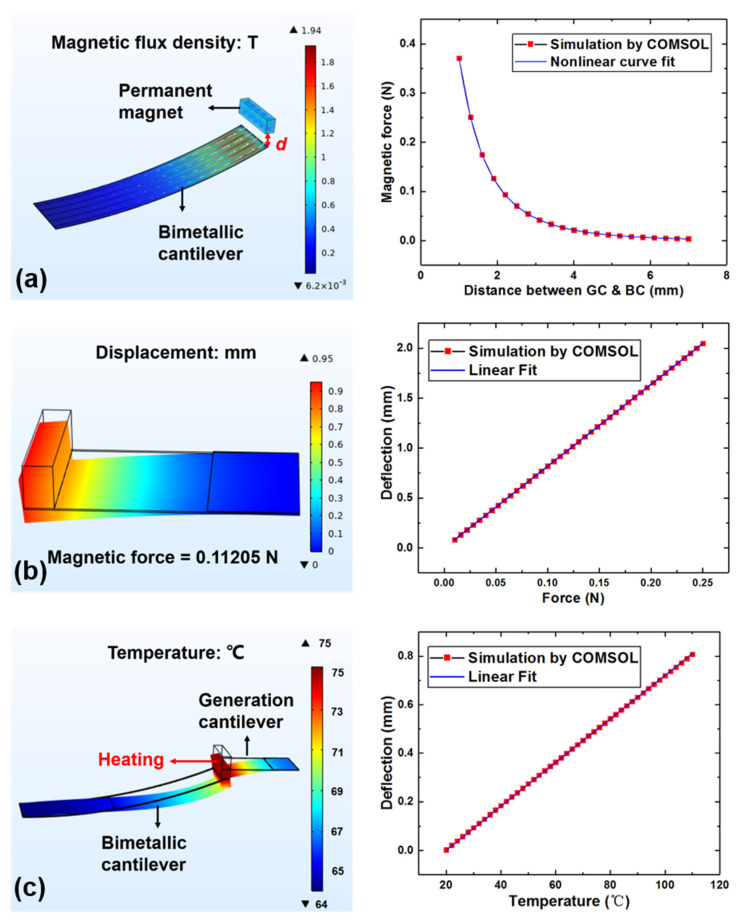
Simulation of the relationship between the temperature threshold and the distance between two stages. (**a**) The magnetic force on the magnetized bimetallic cantilever varies with distance; (**b**) deflection of the generation cantilever when the end of the cantilever is under force; (**c**) the deflection of the generation cantilever changes with the temperature, when the two cantilevers are attracted.

Figure 3a shows the magnetic force of the bimetallic cantilever magnetized by a permanent magnet changes with the distance between two cantilevers. According to the conclusion given in the literature [17], the relationship between magnetic force and distance can be expressed by the fourth power of the distance. Therefore, in this study, the curve obtained in Figure 3a is fitted with the reciprocal of the fourth-order polynomial of the distance *d*, and the coefficient of determination R-Square is 0.99997, indicating the Goodness of Fit meets the demand. Then, the relationship between the magnetic force and the distance between the two stages can be expressed as:

(4) $$F_{mag}=\frac{1}{a_{4}d^{4}+a_{3}d^{3}+a_{2}d^{2}+a_{1}d+a_{0}}\;\;(a_{4}\;=\;-0.09695,\;a_{3}\;=\;-0.18457,\;a_{2}\;=\;2.11676,\;a_{1}\;=\;-0.42567,\;a_{0}\;=\;1.09175)$$

Figure 3b shows the deflection of the generation cantilever with PZT attached based on Hooke’s law. From Figure 3b, the equivalent stiffness *k_eff_* of the generation cantilever can be obtained and expressed as Equation (5):

(5) $$F_{elastic}=k_{eff}\times z\;$$

where *z* is the deflection of the generation cantilever, *k_eff_* = 122.0335 N/m.

When the elastic force is greater than the magnetic force, the generation cantilever is separated from the bimetallic cantilever. Therefore, the situation when the elastic force is equal to the magnetic force can be regarded as the critical point. By combining Equations (4) and (5), the relationship between the deflection of the generation cantilever at critical point and the distance between two stages can be obtained. This can be described by Equation (6):

(6) $$z=\frac{1}{k_{eff}}\times F_{mag}\;$$

Figure 3c shows the simulation results of the deflection of the generation cantilever change with temperature when the generation cantilever and the bimetallic cantilever are in attracted state. The area where the magnet is placed on the generation cantilever is the heated area. COMSOL simulation is conducted for the deflection of the bimetallic cantilever caused by different heat transfer methods shown in Figure 2a. If only the heat conduction is taken into account, the deflection would be 26.73 mm at 70 °C, and when heat convection and heat radiation are included into the simulation, the deflection is 23.40 mm, i.e., they both contribute about 12% to the total deflection. Herein, in order to improve the accuracy of simulation results, the convective heat transfer and radiation heat transfer with the environment are also considered in this model. Taking into account that when the model is working at the ambient temperature, the entire device should not generate any deformation, that is, when *T* = *T_a_*, the deflection *z* should be equal to zero. Meanwhile, it can be seen from the simulation results that the deflection *z* has a linear relationship with the heated temperature *T* which is consistent with the relationship between the deflection of the bimetallic cantilever and the heating temperature [18]. Therefore, the results obtained by simulation can be linearly fitted based on the above conclusions, and finally the relationship can be expressed as Equation (8), where *b* = 0.00902.

(7) $$z=b\times \left(T-T_{a}\right)\;$$

Let Equation (7) be equal to the Equation (6), and the relationship between the temperature threshold and the distance between the two stages can be obtained and expressed as:

(8) $$T=\frac{1}{b}\times \frac{1}{k_{eff}}\times F_{mag}+T_{a}\;$$

**Figure 4:**
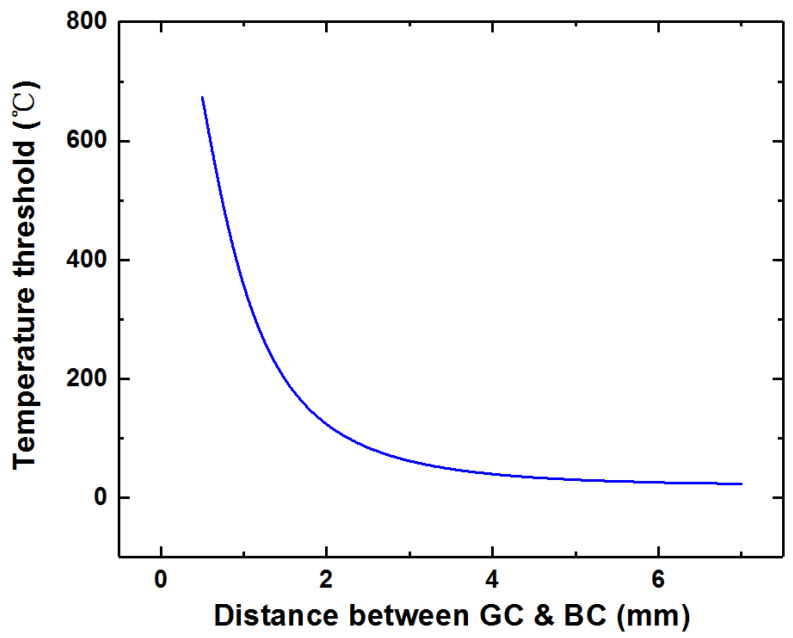
The relationship between the temperature threshold and the distance between the two stages of the energy harvester.

With the above relationship, the temperature threshold of the heat event autonomous monitoring energy harvester can be preset by adjusting the distance between the two stages. Figure 4 is the plot of the relationship between the temperature threshold and the distance between the two stages of the energy harvester according to Equation (8). It can be seen from Figure 4 that when the distance between the two stages is greater than 6 mm, the temperature threshold of the energy harvester is quite close to room temperature 20 °C, which means that there is almost no threshold between the two stages.

## 3. Experimental Results

**Figure 5:**
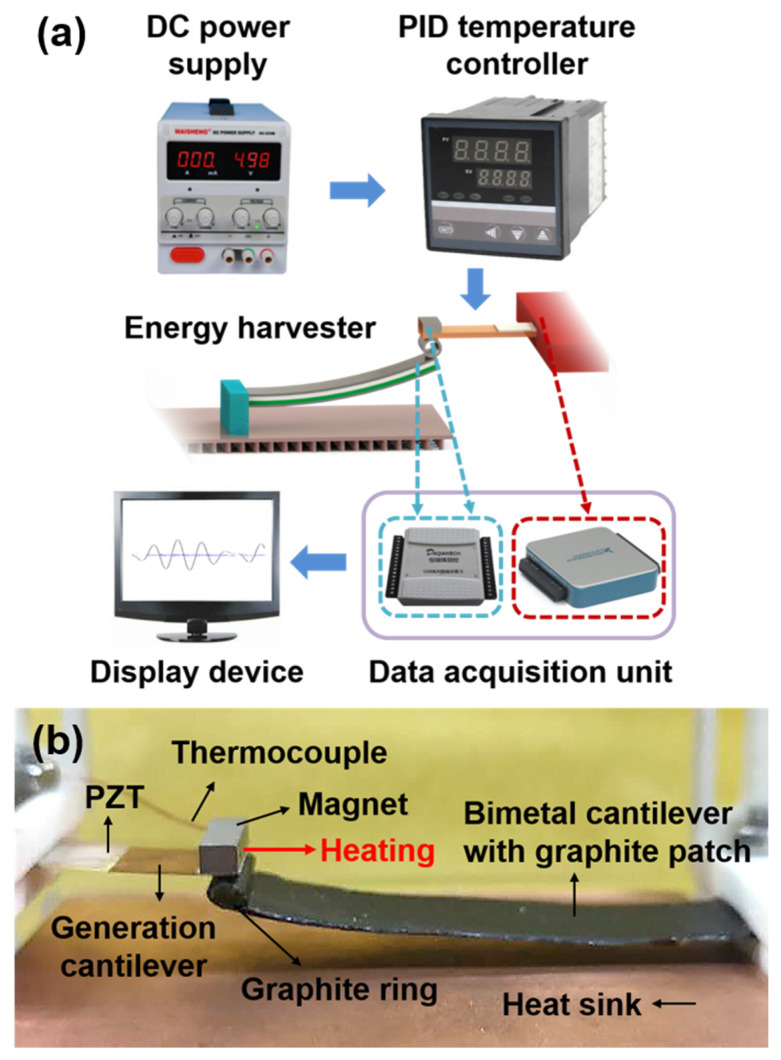
Experimental details of the heat event induced energy harvesting. (**a**) Schematic test setup of the energy harvester and (**b**) physical photo of the energy harvester prototype.

Figure 5a is the test setup of the heat event induced energy harvester. For experimental convenience, the heat source is imitated by input direct current through an attached heating resistor (resistivity of 374 Ω/m, resistance of 200 Ω) on the generation cantilever. The current generated by DC source (Maisheng, MS1510D) can be adjusted by a PID temperature controller. Since it is essential to obtain the power generation data and the temperature data of the device at the same time, two kind of data acquisition units need to be used here. One data acquisition unit (NI USB-6003, 16 bit) with sampling rate as 1000 Hz is used to collect instantaneous analog signals from the generation cantilever, and the other (HRF USB-6009, 16 bit) with sampling rate as 0.5 Hz is used to collect device temperature data in real time. Both kinds of the data acquisition units can display the collected data through the display device. As shown in Figure 5b, the PZT sheet is bonded with the substrate through a kind of conductive silver paste (EPO TEK H20E). The end of the generation cantilever is equipped with a samarium cobalt magnet, whose working temperature can reach up to 350 °C which ensures the stability of the temperature threshold. The heating resistor is fixed near the end of the generation cantilever and an ultra-thin commercial K-type thermocouple with a wire diameter of 0.1 mm is put in touch with the generation cantilever in the same place to measure the heating temperature. The bimetallic cantilever consists of bimetallic layers of a high thermal expansion layer Mn_75_Ni_15_Cu_10_ and a low expansion layer Ni_36_. Thus, the bimetallic cantilever is ferromagnetic and can be magnetized by the magnet and attracted to the end of the generation cantilever. In order to enhance thermal conduction rate of the bimetallic cantilever, the graphite sheets are attached to the double surfaces of the bimetallic cantilever whose in-plane thermal conductivity can reach up to 1200 W/(m·k) which is about three times that of copper. Another ultra-thin commercial K-type thermocouple is placed at the end of the bimetallic cantilever to collect temperature data of the bimetallic cantilever. The graphite ring is placed between the generation cantilever and the bimetallic cantilever to increase the thermal contact area and has the function of adjusting the magnetic force between the two stages. In this way, the temperature threshold of the entire energy harvester can be adjusted by changing the size of the graphite ring. By both systematic analysis and experiment, the temperature threshold of the prototype device is set near 71 °C.

In our experiment, the bimetallic cantilever and the generation cantilever are always attracted together, that is, the generation cantilever never generates electricity, as long as the temperature of the heated generation cantilever is below 71 °C. However, when the temperature reaches or exceeds the threshold temperature of near 71 °C, the generation cantilever will get rid of the attraction and generate electricity through free vibration.

**Figure 6:**
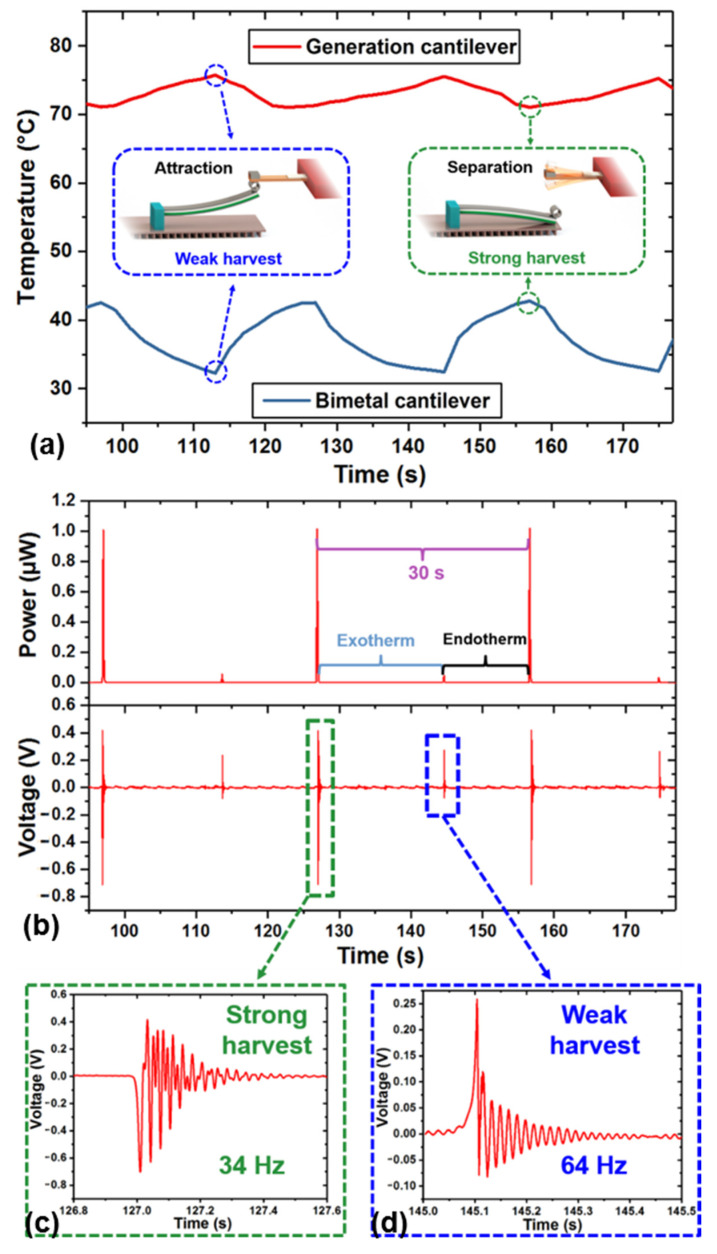
The temperature curves and power generation performance of the energy harvester when triggered at 71 °C. (**a**) The temperatures of the tip of the generation cantilever and the tip of the bimetal cantilever periodically change over time respectively; (**b**) instantaneous output power and output voltage periodically change over time respectively; (**c**) voltage waveform of the strong harvesting process; and (**d**) voltage waveform of the weak harvesting process.

Figure 6a shows the temperature of the heating part and the temperature of the bimetallic cantilever change along with the separation and attraction process between the generation cantilever and the bimetallic cantilever. Initially, the two cantilevers are in attraction state, when the temperature of the bimetallic cantilever rises to no more than 42 °C, the two stages maintain the attracted state. At this time, the temperature of the heating part will drop from 75 °C to 71 °C due to heat flowing to the bimetallic cantilever. When the temperature at the end of the bimetallic cantilever exceeds 42 °C, the two cantilevers will be separated due to deflection of the bimetallic cantilever. In the separation state, the temperature of the bimetallic cantilever gradually decreases because of heat dissipation, and at the same time the temperature of the heating part of the generation cantilever can further rise. When the temperature at the end of the bimetallic cantilever drops to 32 °C and the temperature at the heating part rises to 75 °C, the bimetallic cantilever will restore deformation and return to its original position, and then the energy harvester will get into the attracted state again.

It should be noted that after the separation of the generation cantilever and the bimetallic cantilever, the heating part will be heated up to 75 °C; however, the energy harvester is first triggered at 71 °C, that is, the temperature of the two cantilevers when they are first separated is recognized as the threshold temperature which is 71 °C in this experiment.

Figure 6b shows the periodic power generation characteristics of the energy harvester for heating to 71 °C. A strong harvest process and a weak harvest process are observed in a cycling period of about 30 s. The strong harvest process occurs when the generation cantilever is pulling away from the bimetallic cantilever due to heat absorption, and this causes the generation cantilever to freely resonate and generate electric energy in the form of a one-end-free beam. The weak harvest process corresponds to the generation cantilever impacted and attracted again by the bimetallic cantilever. Due to heat dissipation deformation of the bimetallic cantilever is restored and returned to its original position, and this causes the generation cantilever to vibrate and generate electricity in the form of a buckled beam.

Figure 6c,d are the harvested voltage waveforms during the weak and strong process, respectively. The vibration frequency of the strong process is 34 Hz, which is smaller than 64 Hz during the weak process. The maximum instantaneous peak-to-peak voltage and the maximum instantaneous output power of strong harvest process are 1.12 V and 1.023 μW, respectively. The maximum instantaneous peak-to-peak voltage and the maximum instantaneous output power of weak harvest process are 0.34 V and 0.054 μW, respectively. The event of heating from ambient temperature (20 °C) to 71 °C causes the total instantaneous power generated during the whole cycle (including both the strong and weak harvest processes) reaches to 1.077 μW, and the electric energy generated in one cycle is 0.23 μJ when the temperature gradient is 51 °C.

Under similar temperature gradient, the proposed energy harvester may show lower power generation compared to the works in the literatures of [14,19]. However, our proposed device features the unique temperature-threshold triggered generation function that can be potentially used for autonomous event-driven autonomous sensing applications. In other words, the activity of electricity-power generating itself indicates the occurrence of the monitored object, i.e., the generated electricity also contains the sensing information of the monitored event. Unlike those conventional energy-harvesters, it is not necessary for this device to generate a sufficiently high electric-power to sustain the conventional sensors and their complicated interface-circuit systems. In an alternative method, the generated electric-energy in the proposed device can be only used to support alarming-signal sending out. This event-driven autonomous alarming principle means the device could possibly be used in monitoring WSN applications.

**Figure 7:**
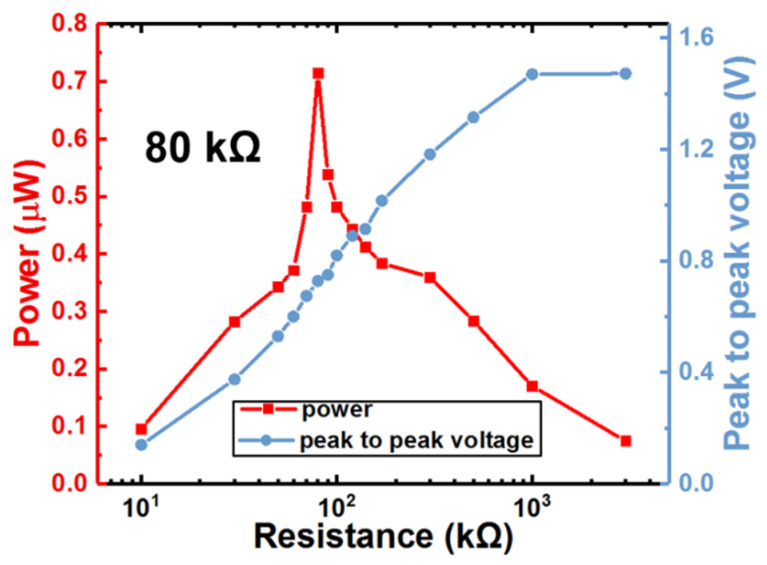
Test results of optimal loading resistance for the strong harvest.

Figure 7 shows the tested peak-to-peak voltage and instantaneous output power of the 71 °C heating induced strong harvest process under different loading resistors. The maximum power of 0.72 μW was reached under the optimal load of 80 kΩ and the open-loop voltage was about 1.47 V. It should be noted that the test results of Figure 6 are all based on the optimal load resistance of 80 kΩ.

**Figure 8:**
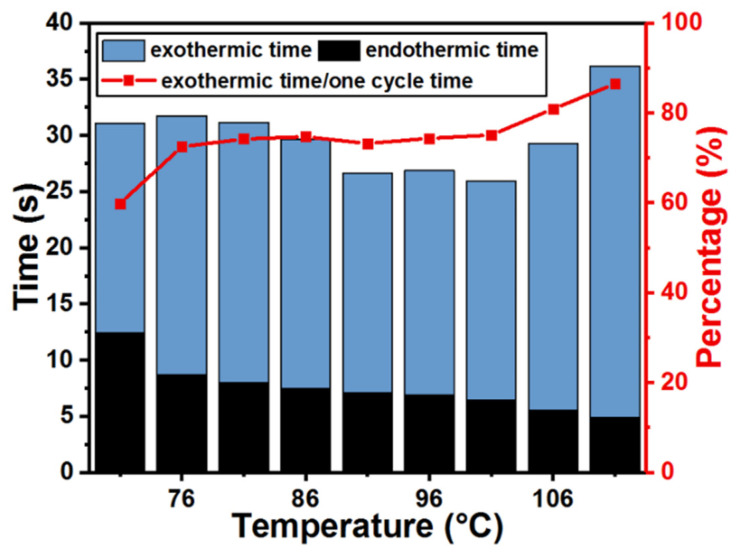
Tested endothermic and exothermic periods versus heating temperature.

Under different heating temperature of the event, Figure 8 shows the tested total generation period, the endothermic time, and the exothermic time which are both marked in Figure 6b after the device is triggered at 71 °C. Along with increasing heating temperature, the endothermic time required for the generation cantilever pulling away from the bimetallic cantilever gradually decreases. Correspondingly, the exothermic time required for the generation cantilever attracted by the bimetallic cantilever and the proportion of exothermic time to the entire generation period generally remain stable for the temperature range of 76 °C to 101 °C. Exceeding 101 °C however, the exothermic time greatly increases, which leads to an increase of the total time period of one cycle, possibly due to more heat needs to be dissipated when the working temperature exceeding 101 °C.

## 4. Conclusions

A thermal energy harvester temperature threshold triggered generation for heat event autonomous monitoring is proposed and implemented. The proposed harvester can simultaneously output cyclic generation of electric energy and temperature-threshold triggered event information. In future work, the device would be further optimized to transmit the monitored heat event information by using the generated electric, thereby realizing self-powered WSN nodes for autonomous alarming.

## Figures and Tables

**Table 1:** Thermal conductivity and emissivity of thermal conductive materials.

Materials	Thermal Conductivity W/(m·K)	Emissivity
Bimetal	10.9	0.25
Graphite sheet	1200	0.85
Copper foil	400	0.3
Gallium-based alloy	120	0.5

**Table 2:** The material properties and geometric parameters of the energy harvester.

Material Properties	Parameters
Bimetallic cantilever material	Mn_75_Ni_15_Cu_10_ Ni_36_
Generation cantilever material	beryllium bronze
Magnet material	samarium cobalt
Maximum working temperature of the magnet	350 °C
Bimetallic cantilever thickness	0.1 mm
Bimetallic cantilever length	47 mm
Bimetallic cantilever width	10 mm
Generation cantilever thickness	0.1 mm
Generation cantilever length	18 mm
Generation cantilever width	10 mm
Magnet length	10 mm
Magnet width	2 mm
Magnet height	3 mm
PZT thickness	0.06 mm
PZT length	6 mm
PZT width	10 mm
Distance between the two cantilevers	2.5 mm
Graphite sheet thickness	0.07 mm

## References

[1] Boughaleb J., Arnaud A., Cottinet P.J., Monfray S., Gelenne P., Kermel P., Quenard S., Boeuf F., Guyomar D., Skotnicki T. (2015). Thermal modelling and optimization of a thermally matched energy harvester. Smart Mater. Struct..

[2] Olsson R.H., Bogoslovov R.B., Gordon C. Event Driven Persistent Sensing: Overcoming the Energy and Lifetime Limitations in Unattended Wireless Sensors. Proceedings of the 15th IEEE Sensors Conference.

[3] Kang S.B., Kim J.H., Jeong M.H., Sanger A., Kim C.U., Kim C.M., Choi K.J. (2019). Stretchable and colorless freestanding microwire arrays for transparent solar cells with flexibility. Light-Sci. Appl..

[4] Liu X., Ma J., Wu X., Lin L., Wang X. (2017). Polymeric nanofibers with ultrahigh piezoelectricity via self-orientation of nanocrystals. ACS Nano.

[5] Wang Y., Shi Y., Mei D., Chen Z. (2018). Wearable thermoelectric generator to harvest body heat for powering a miniaturized accelerometer. Appl. Energy.

[6] Ravi K., Shashank P. (2018). A review on low-grade thermal energy harvesting: Materials, methods and devices. Materials.

[7] Yang S.M., Wang S.H. (2020). Development of a thermoelectric energy generator with double cavity by standard cmos process. IEEE Sens. J..

[8] Hinterleitner B., Knapp I., Poneder M., Shi Y., Müller H., Eguchi G., Eisenmenger-Sittner C., Stöger-Pollach M., Kakefuda Y., Kawamoto N. (2019). Thermoelectric performance of a metastable thin-film Heusler alloy. Nature.

[9] Hao F., Qiu P., Tang Y., Bai S., Xing T., Chu H.S., Zhang Q., Lu P., Zhang T., Ren D. (2016). High efficiency Bi2Te3-based materials and devices for thermoelectric power generation between 100 and 300 degrees C. Energy Environ. Sci..

[10] Hunter S.R., Lavrik N.V., Rajic S., Datskos P.G. (2012). Review of pyroelectric thermal energy harvesting and new MEMs based resonant energy conversion techniques. Proceedings of the Conference on Energy Harvesting and Storage: Materials, Devices, and Applications III.

[11] Leng Q., Chen L., Guo H., Liu J., Liu G., Hu C., Xi Y. (2014). Harvesting heat energy from hot/cold water with a pyroelectric generator. J. Mater. Chem. A.

[12] Kishore R.A., Priya S. (2018). A review on design and performance of thermomagnetic devices. Renew. Sustain. Energy Rev..

[13] Chun J., Song H.C., Kang M.G., Kang H.B., Kishore R.A., Priya S. (2017). Thermo-Magneto-Electric Generator Arrays for Active Heat Recovery System. Sci. Rep..

[14] Deepak K., Varma V.B., Prasanna G., Ramanujan R.V. (2019). Hybrid thermomagnetic oscillator for cooling and direct waste heat conversion to electricity. Appl. Energy.

[15] Song H.C., Maurya D., Chun J., Zhou Y., Song M.E., Gray D., Yamoah N.K., Kumar D., McDannald A., Jain M. (2017). Modulated Magneto-Thermal Response of La_0.85_Sr_0.15_MnO_3_ and (Ni_0.6_Cu_0.2_Zn_0.2_)Fe_2_O_4_ Composites for Thermal Energy Harvesters. Energy Harvest. Syst..

[16] Waske A., Dzekan D., Sellschopp K., Berger D., Stork A., Nielsch K., Fähler S. (2019). Energy harvesting near room temperature using a thermomagnetic generator with a pretzel-like magnetic flux topology. Nat. Energy.

[17] Vokoun D., Beleggia M., Heller L., Sittner P. (2009). Magnetostatic interactions and forces between cylindrical permanent magnets. J. Magn. Magn. Mater..

[18] Timoshenko S. (1925). Analysis of bi-metal thermostats. J. Opt. Soc. Am. Rev. Sci. Instrum..

[19] Gao Q., Wang W., Lu Y., Cai K., Li Y., Wang Z., Wu M., Huang C., He J. (2021). High Power Factor Ag/Ag_2_Se Composite Films for Flexible Thermoelectric Generators. ACS Appl. Mater. Interfaces.

